# Supplementary material for: Deep neural networks to recover unknown physical parameters from oscillating time series
Source: PLoS One. 2022 May 13;17(5):e0268439. doi: 10.1371/journal.pone.0268439 (PMC9106171; doi:10.1371/journal.pone.0268439)
Supplement: S1 Appendix — (PDF) [file pone.0268439.s001.pdf]

A Supplementary information

A.1 DNN architecture implementation

The pseudo-code architectures of the *Encoder*, *Regressor*, *Decoder*, and unified DNN, as implemented in Python (Keras - Tensorflow), are given below in addition to the custom weighted-loss function.

```
bottleneck_dim = 64 # Output dimension of the Encoder
latent_dim     = 7  # Output dimension of the Regressor
signal_length  = 512

## ENCODER subDNN
input_shape = (signal_length ,1)
i = Input(shape=input_shape)
x = Conv1D(64, kernel_size=64, activation='relu' ,padding='same')(i)
x = MaxPooling1D(4, padding='same')(x)
x = Conv1D(64, kernel_size=32, activation='relu' ,padding='same')(x)
x = MaxPooling1D(4, padding='same')(x)
x = Flatten()(x)
x = Dense(128, activation='relu', kernel_initializer=''he_uniform'')(x)
bottleneck = Dense(64, activation='relu', kernel_initializer=''he_uniform'')(x)

encoder = Model(i, bottleneck)

## REGRESSOR subDNN
input_shape = (bottleneck_dim ,1)
r_i = Input(shape=input_shape)
x = Reshape((bottleneck_dim ,1))(r_i)
x = Conv1D(64, kernel_size=64, activation='relu' ,padding='same')(x)
x = MaxPooling1D(4, padding='same')(x)
x = Conv1D(64, kernel_size=32, activation='relu' ,padding='same')(x)
x = MaxPooling1D(4, padding='same')(x)
x = Conv1D(64, kernel_size=32, activation='relu' ,padding='same')(x)
x = Flatten()(x)
x = Dense(256, activation='relu', kernel_initializer=''he_uniform'' )(x)
x = Dense(128, activation='relu', kernel_initializer=''he_uniform'' )(x)
x = Dense(64,  activation='relu', kernel_initializer=''he_uniform'' )(x)
latent = Dense(latent_dim)(x)

regressor = Model(r_i, latent)

## DECODER subDNN
input_shape = (bottleneck_dim+latent_dim ,1)
d_i   = Input(shape=input_shape)
x = Dense(128, activation='relu', kernel_initializer=''he_uniform'')(d_i)
x = Reshape((128,1))(x)
x = Conv1D(64, kernel_size=32, activation='relu', padding='same')(x)
x = MaxPooling1D(2, padding='same')(x)
x = UpSampling1D(4)(x)
x = Conv1D(64, kernel_size=32, activation='relu', padding='same')(x)
x = MaxPooling1D(2, padding='same')(x)
x = UpSampling1D(4)(x)
x = Conv1D(64, kernel_size=32, activation='relu', padding='same')(x)
x = MaxPooling1D(2, padding='same')(x)
x = UpSampling1D(2)(x)
decoded = Conv1D(1, kernel_size=32, activation='sigmoid', padding='same')(x)

decoder = Model(d_i, decoded)

## Unified DNN model
concat = Concatenate()([encoder(i),regressor(encoder(i)) ])
ae_outputs = decoder(concat)
flatten_ae_outputs = Reshape((signal_length,))(ae_outputs)
concat2 = Concatenate()([flatten_ae_outputs, regressor(encoder(i)) ])
DNN_outputs = concat2
```

```
DNN = Model(i, DNN_outputs)

## CustomLoss function
def customLoss(yTrue,yPred):
    latentSize = 7
    SignalSize = 512
    beta = 0.001
    mseSignal = K.square(yTrue[:, 0:SignalSize] - yPred[:, 0:SignalSize])
    mseSignal = K.abs(mseSignal)
    mseSignal = K.sum(mseSignal, axis=-1)
    mseSignal = mseSignal/SignalSize

    mseLatent = K.square(yTrue[:, SignalSize:] - yPred[:, SignalSize:])
    mseLatent = K.abs(mseLatent)
    mseLatent = K.sum(mseLatent, axis=-1)
    mseLatent = mseLatent/latentSize

    weighted_mse = (1-beta)*mseSignal + beta*mseLatent

    return weighted_mse


DNN.compile(optimizer = 'ADAM', loss = customLoss)
DNN.summary()
```

A.2 Supplementary figures

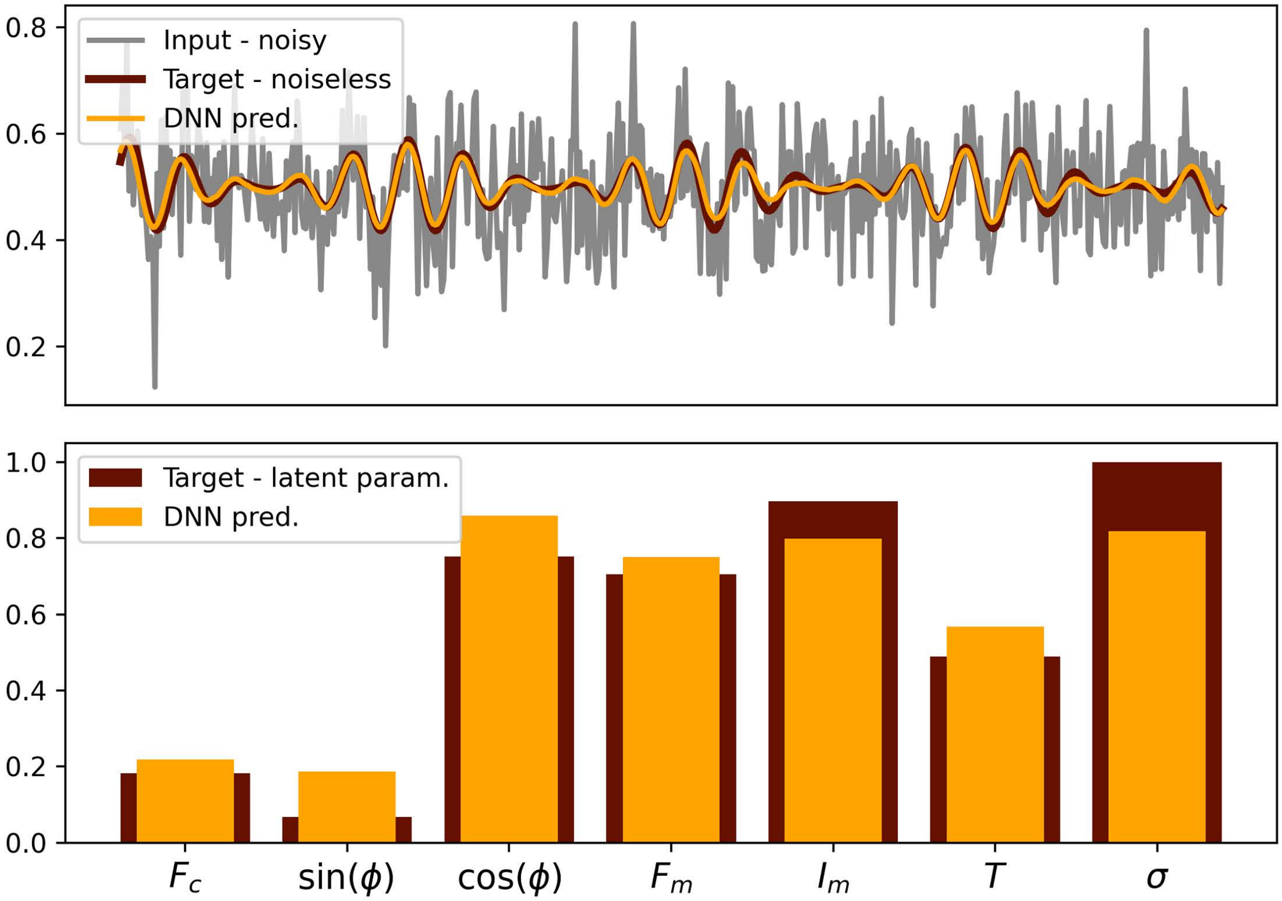

Fig S1. Example of DNN prediction using a noisy AM-sine wave input. The DNN was trained only on AM-sine waves samples. **Top**: Noisy input and *Decoder* denoised prediction. **Bottom**: True latent parameter targetsand *Regressor* prediction. The phase ϕ is mapped to two separate latent variables to accommodate for phase periodicity during loss computation.

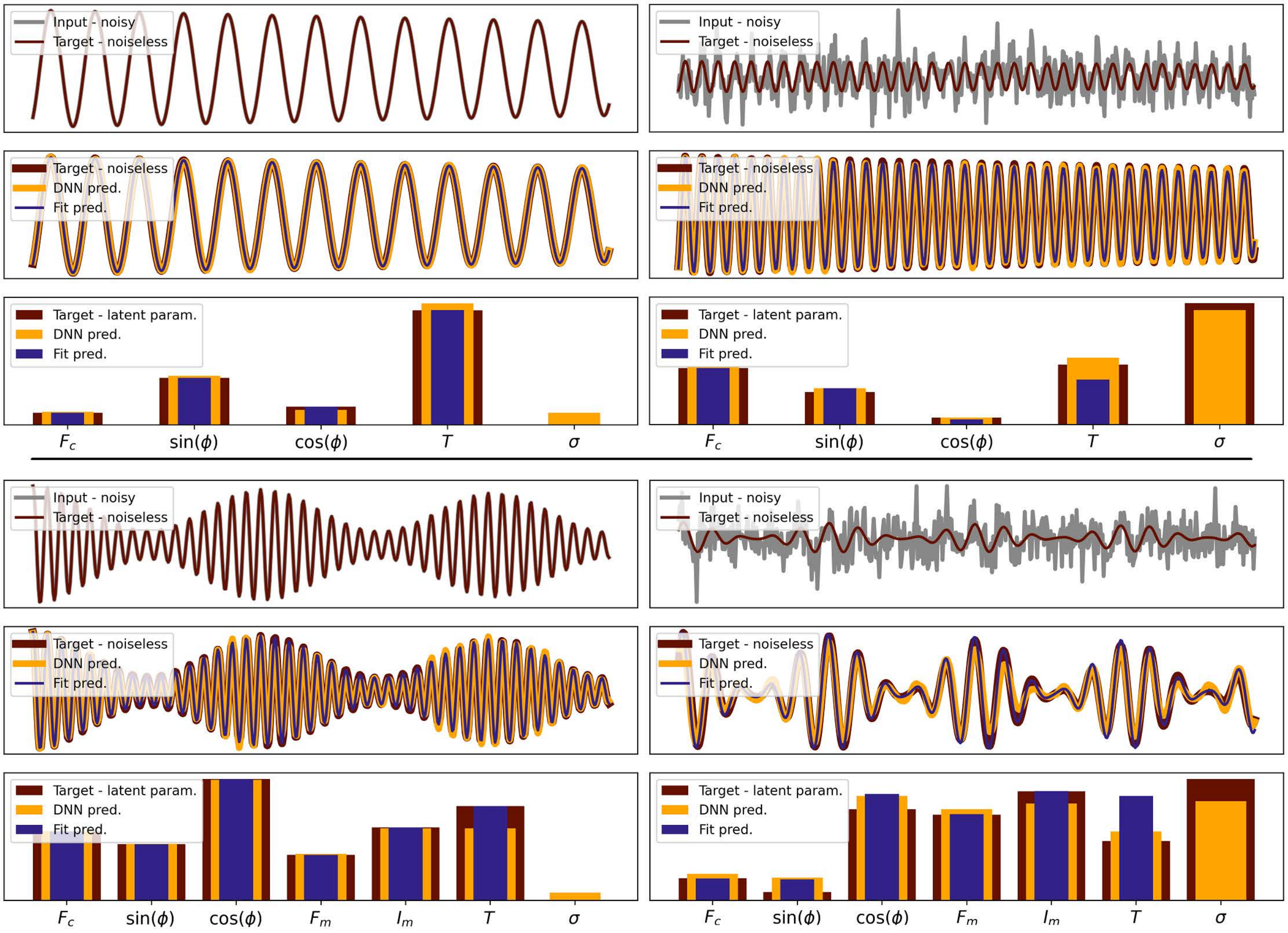

Fig S2. Example of minimum (left) and maximum noise (right) sine wave (top) and AM-sine wave (bottom) samples. DNN and LS-fits denoised and latent parameters predictions are shown below the respective inputs.

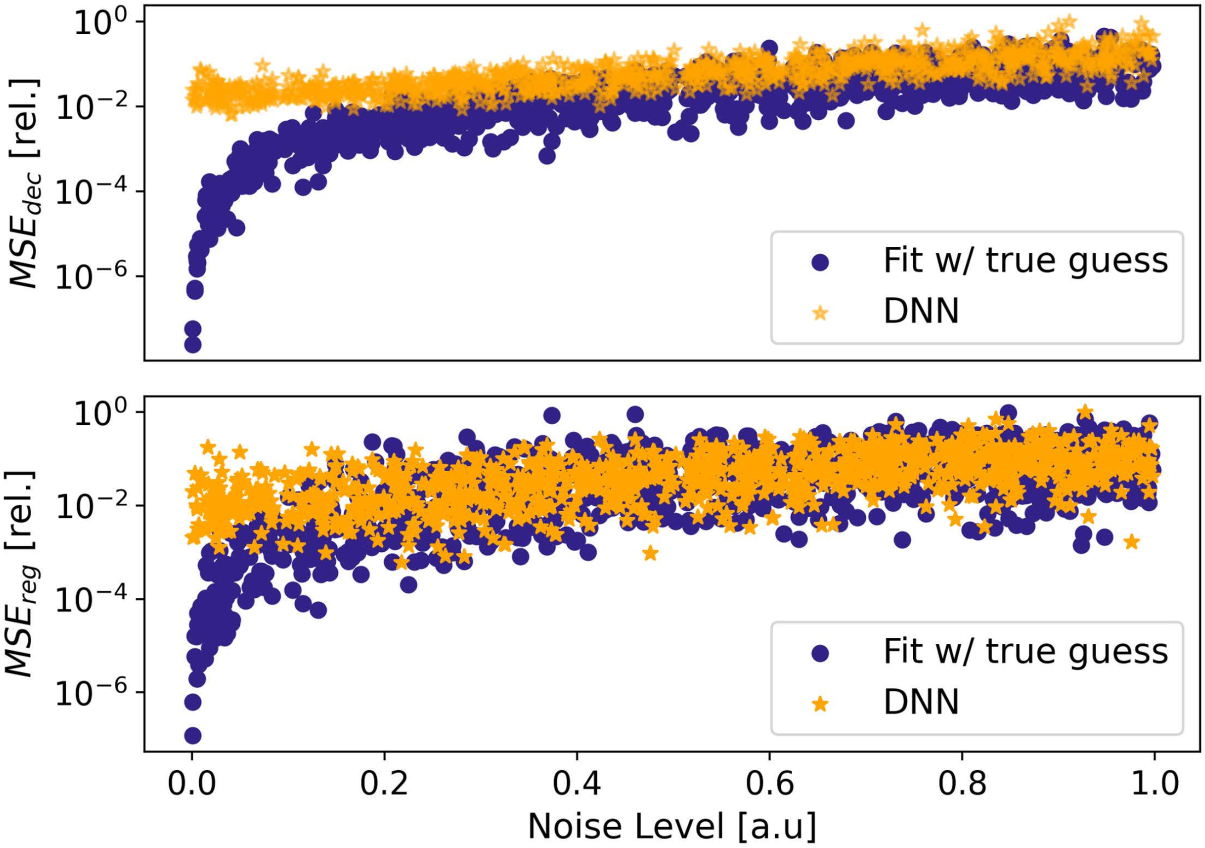

Fig S3. Comparison of DNN post-training performance to LS-fits with true latent-parameters initial guesses for 1000 random AM-sine wave from the test set (unseen during training). The denoising (MSE_{dec}, top) and latent-parameters relative regression losses (MSE_{reg}, bottom) are sorted by increasing noise levels. The denoising (MSE_{dec}, top) and latent-parameters regression losses (MSE_{reg}, bottom) are sorted by increasing noise levels. The DNN was trained only on AM-sine waves samples. See Fig. 3 for MSE_{reg} and MSE_{dec} computation methods. The LS-fit with true initial guesses vastly outperforms the DNN for low-noise signals but both systems reach similar performance for high-noise.

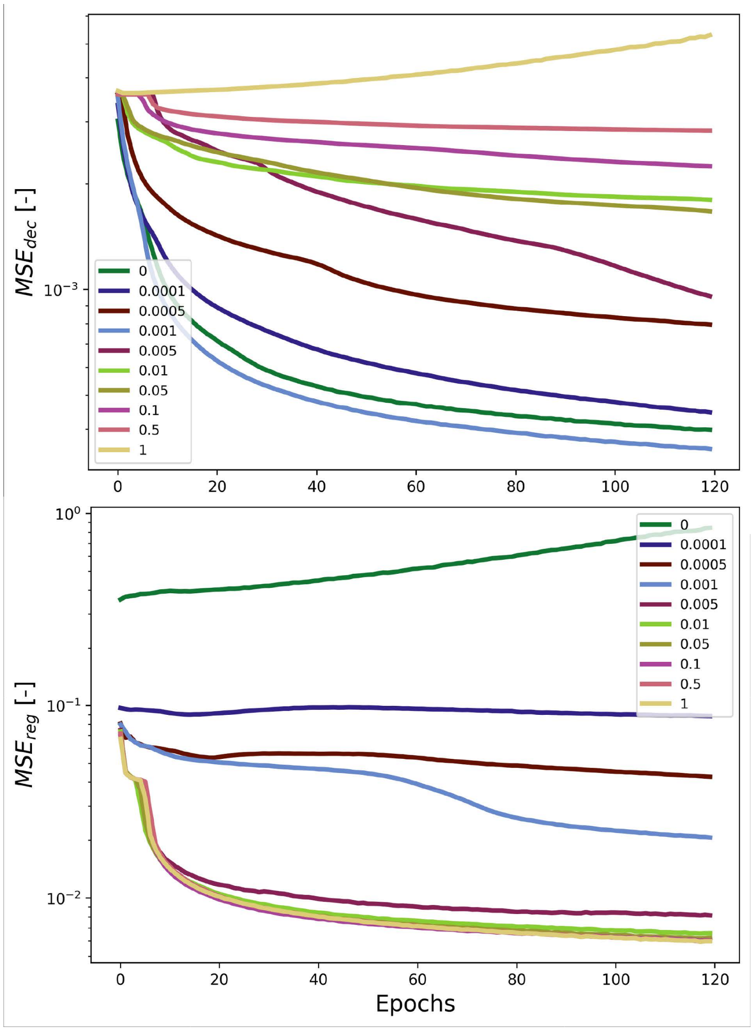

Fig S4. Validation loss during training for various values of β. **Top**: signal denoising losses. **Bottom**: latent-parameters regression losses. Training is performed on 12 training sets of $100'000$ randomly generated FM-sine waves for 10 epochs. For clarity, validation-loss is displayed after each individual training set backpropagation.
