# Supplementary material for: Deep neural networks to recover unknown physical parameters from oscillating time series
Source: PLoS One. 2022 May 13;17(5):e0268439. doi: 10.1371/journal.pone.0268439 (PMC9106171; doi:10.1371/journal.pone.0268439)
Supplement: Attachment — Submitted filename: Response to Reviewers.pdf [file pone.0268439.s002.pdf]

Response to the reviewers : Deep Neural Networks to Recover Unknown Physical Parameters from Oscillating Time Series

Antoine Garcon, Julian Vexler, Dmitry Budker, Stefan Kramer

March 29, 2022

Dear editors of PLOS One,

We are grateful to the reviewers for their insightful comments and suggestions. We have been able to take actions on every items suggested by the reviewers who enabled us to improve the manuscript. Changes are highlighted within the manuscript. The following contains is a point-by-point response to the reviewers suggestions.

Reviewer 1, comment 1/8:

In Fig 2, two latent parameters are used for learn about ϕ. How the trigonometric relationship between these components is ensured? It is, how is it ensured in the model that $sin^2\phi + cos^2\phi = 1$?

The Pythagorean identity was not used as a constraint during this work. After investigation, we measured that this identity was violated on the same order of magnitude as the error on the phase prediction itself. Nonetheless, we thank the reviewer for this suggestion and now discuss the potential use of latent parameters constraints in the Conclusion section of this article, in addition to discussing the reviewer's idea to use recurrent neural networks for arbitrarily long signals (see reviewer 1, comment 8/8). The said paragraph now reads:

"Further performance improvements could also be obtained by using known constraints on the latent-parameters. In the case of this work, one could encode the Pythagorean identity to ensure the two-point phase prediction satisfies $\cos(\phi)^2 + \sin(\phi)^2 = 1$. Other constraints may include the expected allowed ranges of each latent variables. This is typically done by adding a cost to the loss function used during training. In our work, because the Pythagorean identity was violated on the same order of magnitude as the phase-prediction error, we did not implement such constraint but we nonetheless mention this as a technical possibility. Implementing constraints has been shown to improve performance and would further reduce the black-box character of DNNs by adding specific domain-knowledge to the model [22,23]."

Reviewer 1, comment 2/8:

How is MSE_{reg} computed? A detailed description in text is required.

The computation technique was indeed not described. A short sentence has been added above Eq. 1 to clarify :

"The latent-parameter regression loss, MSE_{reg}, and signal-denoising losses, MSE_{dec}, are computed simultaneously. MSE_{reg} is the mean squared error between the DNN predictions and true latent parameters, while MSE_{dec} is the mean squared error between the denoised signal prediction and the noiseless signal."

Reviewer 1, comment 3/8:

β parameter is assumed to help in bias control of the model (Pg 5, last paragraph). Please give detailed description of how this is achieved by tuning the β parameter.

We now include a short description of the tuning of β in the main text:

"The value of β is determined by performing a grid search over the $[0, 1]$ range: We train a new instance of the DNN for varying values of β. The best value (yielding the lowest overall loss and low bias towards any of the tasks, determined on a validation set) is maintained for further training of the DNN. Details on this training procedure and search for optimal values of β are given in the Methods section of this article."

A more detailed discussion of the effect of the bias parameter is included in the Methods section, supported by Fig.S4 & Fig.6, which illustrate the effect of this hyper-parameter during and after training.

Reviewer 1, comment 4/8:

For LS-fit, please provide the exact model used.

The model used were the exact generating functions for the AM, FM and monochromatic sinewave, given in the Materials and Method. This was not clearly stated in section 2.3 which now reads:

"When performing the LS-fit, the input data is the noisy signals and the model function consists of the exact noiseless data-generating functions, given in Section 4.1."

Reviewer 1, comment 5/8:

How good is LS-fit performance with random initialization followed by gradient descent training (similar to DNNs)?

Since both reviewers 1&2 had similar concerns about LS-fits initialization and performance, we addressed them together by entirely rewriting section 2.3. We now indicate within section 2.3:
1- The poorness of the outcome for LS-fit with random initialization (reviewer 1 comment 5)
2- A description of state-of-the-art signal processing tools to recover latent parameters (reviewer 2 comments 1 & 2)
3- We explain that the DNN-assisted fit can be an interesting technique to automate searching the space of initial guesses prior to LS-fits (reviewer 2 comment 2)

Reviewer 1, comment 6/8:

It is not clear why deep learning is better than LSfit method for the problems discussed in the manuscript. One of the points that is consistently repeated is that LSfit is required to know an initial point. However the same goes with DNNs as well. Please clarify how a randomly initialized DNN is better than LSfit in this case?

We feel that our presentation needed improvement, in particular of the DNN and LS-fit initializations. Please note the following:

a) On the DNN and LS-fits initialization: In this work, the DNN is always initialized at random prior to training (by assigning random weights to each neuron). LS-fits, however, require "good" initial points, typically referred to as "initial guesses", which must be close to the true latent parameters.

b) On the potential advantage of using the DNN: The advantage of the neural network approach is then to automate the exploration of the initial space performed during LS-fits (which is replaced by training the DNN on a general dataset).

These statements are made clearer in section 2.3:

"These results show that our architecture can be a good alternative to LS-fits for time-series analysis. First, the DNN reaches acceptable performance when benchmarked to standard LS-fits with true guesses, while needing no initial guesses (the DNN being initialized randomly prior to training). Moreover, the complex task of exploring the initial-guesses space for each sample during LS-fit is no longer needed when employing the DNN. This can automate and accelerate data processing."

In addition, we modified the legend of Fig. 4 such as to make clearer the fact that the DNN is used to assist LS-fits initial guesses exploration. We hope that this will avoid confusion about the DNN initialization and advantages. It was indeed misleading before.

Reviewer 1, comment 7/8:

The proposed model is designed to work for only specific length sequences (512 in the presented experiments). However, with the given sampling specification, this may under-sample the signal which might result in missing the signal. Please provide a description on the limitations of sampling/network input and how this could be handled.

We ensure the signals is never over/undersampled by adjusting the range of allowed signal frequencies during data generation. We modified Section 2.1 to now state this clearly:

"Before each sample generation, the latent parameters are randomly and uniformly sampled within their respective allowed range (see Methods). The range of F_c ensures the carrier frequency remains well within the Fourier and Nqyist limits such that no over- or under-sampling occurs. The modulation amplitude range ensures the majority of the signal's power remains in its first sidebands and carrier."

Moreover, the experiment can be run with any signal length by adjusting the number of input neurons of the encoder and output neurons of the decoder. In addition, we added a short statement concerning this topic in the conclusion of the article (see also reviewer's suggestion below).

Reviewer 1, comment 8/8:

One of the ways to extent the architecture to handle arbitrary length sequences is to use recurrent neural network based encoder-decoder framework. It may be useful for the reader if a description pointing towards this direction is included.

We thank reviewer 1 for this suggestion, which we added in the conclusion section of the article:

"[...]. Moreover, the system can be employed for signals of various lengths by adjusting the number of input and output neurons of the $Autoencoder$*. For signals of arbitrary and varying length, or for online applications, a recurrent neural network architecture could also be implemented [21]. In both cases however, the end user must remain aware of the possibility of over- and under-sampling signals. Indeed, in the case of this work, the frequency resolution is limited by the number of input and output neurons of the* $Autoencoder$*. Moreover, the memory of a recurrent neural network is always practically finite, regardless of the signal length. The user must then adjust the allowed signal frequencies by restricting the latent parameters' ranges employed during data generation."*

Reviewer 2, general comment:

The authors propose a deep learning model which combines an autoencoder and a regressor to estimate the latent parameters from an oscillating time series. The proposed idea where the output of the DNN is used as an initial estimate for Ls-fit is interesting. The authors' extension to estimating the parameters without knowing the exact data generation process was also impressive. Please find detailed comments listed below.

We thank reviewer 2 for their kind comment.

Reviewer 2, comment 1/4:

My major concern is the lack of literature review regarding how the latent parameter recovery is performed using traditional signal processing tools; in particular, the state-of-the-art method for this specific task.

Typically this task requires expertise on the side of the trained engineer or physicist. A search over the initial guesses is manually performed by repeating LS-fits with different initial guesses. We now describe the typical approach to time-series regression in section 2.3 which has been entirely rewritten:

"Fitting oscillating time series using LS-fits is notoriously difficult because the MSE is in general a non-convex function of the latent parameters. As a result, poorly initialized LS-fits often remain trapped in local minima. Consequently, the quality of the LS-fit is highly dependent on the initial guesses in addition to the noise. For the same reason, random LS-fit initialization yields poor results with very high variability [6].
In any real-world setting, the user must perform additional preprocessing work or use prior information to find initial guesses leading to the global minima. When latent parameters can only exist within finite ranges (e.g. the signal phase must be constrained within $[0, 2\pi[$), a typical method involves performing a grid search over the space of initial guesses [16]. The LS-fit is repeatedly performed by using different values within the ranges. The LS-fit with the lowest final loss, is then assigned to be the best and its predictions are kept. Other methods involve performing a first estimation of the true latent parameters prior to performing the LS-fit (e.g. the signal's carrier frequency can be approximated by counting the numbers of local maxima within the time windows or by finding the location of the maximum value of the signal's Fourier transform). Therefore, the complexity, runtime and usertime of LS-fits is highly dependent the amount of samples to analyze and the types of signals at hand."

Reviewer 2, comment 2/4:

The authors provide all their comparisons with LS-fit where the true parameters are known. As pointed out, this is not a fair comparison as the true parameters are generally unknown. The authors are encouraged to provide comparisons with methods that do not need to know the true parameters. If such a method involves a search over the parameter space followed by LS-fit, it is recommended that the authors compare complexity (in addition to performance).

The reviewer is correct in stating that most methods involve a search over the initial guesses space, followed by an LS-fit (see also reviewer's previous suggestion above). The complexity of such searches is entirely dependent on the situation at hand. Indeed, the complexity of the task would depend on the types of signals to analyze, possible access to initial guesses and most importantly on the number of samples to analyze.
To address this comment, we implemented an LS-fit algorithm to perform a search over the initial space and compared its runtime to that of the DNN. The algorithm employs a combination of expert-knowledge to estimate some latent parameters, and performs a grid search for parameters that are more difficult to access. The average runtime for a single signal is on the order of 1 s (LS-fit gradient descent included). Thus, fitting an entire dataset (100000 signals) yields a runtime on the order of one day. On the other hand, training the DNN took approximately one day and inference on the entire dataset takes only a few seconds. Thus, for a large number of samples or for complex data-generating models, employing the DNN as a regression tool could prove faster than repeating LS-fits.

For this reason we now state in section 2.3:

"[...]. In order to compare the runtime performance of the DNN to real-world LS-fits (for which the initial guesses are unknown), we implemented an algorithm to perform a search over the initial space prior to the LS-fits. The samples are taken from the AM-sinewave validation sets. We first estimate the carrier frequency by finding the location of the power spectral density maximum. The signal phase is estimated by computing its gradient over the first half period. All other latent parameters are estimated by performing a grid search over their allowed ranged. The LS-fits function is then run for all initial guess combinations. Parameters yielding the lowest final loss are kept for the final LS-fit.
The average runtime for a single signal is on the order of 1 s (LS-fit gradient descent included). Thus, fitting an entire dataset ($100'000$ signals) yields a runtime on the order of one day. On the other hand, training the DNN took approximately one day and inference on the entire dataset takes only a few seconds.
As a result in some cases, employing the DNN could prove faster and less complex. Indeed, initial guess exploration prior to LS-fits linearly depends on the number of samples to analyze and their length, whereas training DNN does not. Moreover, LS-fits performance and initial-guess exploration is highly dependent on the complexity and number of latent parameters of the data-generating model, which is not the case for DNNs.
These results show that our architecture can be a good alternative to LS-fits for time-series analysis. First, the DNN reaches acceptable performance when benchmarked to standard LS-fits with true guesses, while needing no initial guesses (the DNN being initialized randomly prior to training). Moreover, the complex task of explorating the initial-guesses space for each individual sample being replaced by training the DNN, can automate and accelerate data processing."

Reviewer 2, comment 3/4:

The authors state in their abstract (and introduction) that physicists require quantitative information regarding their systems which is not provided by neural networks (which acts as a black box model). However, they do not address this issue in their work. The proposed method is also obtained from abstract data and does not provide interpretable insights regarding the learnt model.

We feel that our method, while not fully resolving this issue, is still a step toward the right direction. Indeed, we first employ a neural network to fully recover latent parameters of time-series, in addition to denoise the actual signals. While being far from a fully explainable AI or a Physics learner, this method still provides additional quantitative information (latent parameters estimation) than simply outputting a denoised DNN prediction.
Moreover, the use of DNN in time-series regression is typically frowned upon in physics due to the complication of accessing point-estimation uncertainty. Here, we propose to use the DNN to inform LS-fits initial guesses, thus enabling access to a covariance matrix and uncertainty estimation.
We do feel that this was not clearly stated in section 2.4, which was modified by adding the following:

"Because the DNN predictions are always within the venicity of the true parameters, almost all DNN-assisted LS-fits converge to optimal solutions. In settings when the initial guesses are unknown or samples are numerous, the user can initially train the DNN on synthetic data and use it for DNN-assisted fits. As the latter performs optimally regardless of the noise level, this enables fast and accurate analysis of large datasets by automating the initial guesses exploration. Moreover, in addition to accelerated data processing, the use of DNNs to assist LS-fits can partially mitigate the black-box character of DNNs. Indeed, this conjunction use of DNNs and LS-fits enables access to point-prediction uncertainties, typically in the form of covariance matrices, which DNNs typically lack [17-19]."

Reviewer 2, comment 4/4:

A few details in results seem to be missing. For example, Figures 5 (Top) shows the noisy input signal along with the predicted parameters. Indicate if this is the best or the average behavior out of the 100000 samples.

The signal was selected at random among the 100000 samples. Performance for this particlar sample was averaged. This is now clearly stated in the figure caption.
